# Analysis and Prediction of Temperature Using an Artificial Neural Network Model for Milling Glass Fiber Reinforced Polymer Composites

**DOI:** 10.3390/polym16233283

**Published:** 2024-11-25

**Authors:** Paulina Spanu, Bogdan Felician Abaza, Teodor Catalin Constantinescu

**Affiliations:** 1 Manufacturing Engineering Department, National University of Science and Technology POLITEHNICA Bucharest, 060042 Bucharest, Romania; 2 General Nursing Assistance Department, National University of Science and Technology POLITEHNICA Bucharest, 060042 Bucharest, Romania; teodor.constantinescu@upit.ro

**Keywords:** milling, temperature, polymer composite, artificial neural network, LabVIEW

## Abstract

Milling parts made from glass fiber-reinforced polymer (GFRP) composite materials are recommended to achieve the geometric shapes and dimensional tolerances required for large parts manufactured using the spray lay-up technique. The quality of the surfaces machined by milling is significantly influenced by the temperature generated in the cutting zone. This study aims to develop an Artificial Neural Network (ANN) model to predict the temperature generated when milling GFRP. The ANN model for temperature prediction was created using a virtual instrument developed in the graphical programming language LabVIEW. Predicting temperature is crucial because excessive heat during milling can lead to several issues, such as tool wear and thermal degradation in the polymer matrix. The temperature in the tool–workpiece contact surface during the milling process was measured using a thermography technique with a ThermaCAM SC 640 camera (provided by FLIR Systems AB, Danderyd, Sweden), and the data were analyzed using the ThermaCAM Researcher Professional 2.8 SR-2 software. Experimental research shows that the cutting speed has a much more significant effect on the temperature in the cutting zone compared to axial depth of cut and feed speed. The maximum temperature of 85.19 °C was measured in the tool–workpiece contact zone during machining at a cutting speed of 75.39 m/min, a feed rate of 250 mm/min, and an axial depth of cut of 12 mm. This temperature rise occurred due to the larger contact area and heightened friction resulting from the abrasive characteristics of the reinforcement material.

## 1. Introduction

Products made of glass fiber-reinforced polymer (GFRP) materials are used across various industrial sectors, covering about 90% of the global polymer composites market [1,2]. These materials are increasingly preferred in the aerospace, maritime, and automotive industries due to their corrosion resistance, light weight, and exceptional mechanical properties [3,4,5]. Therefore, products designed for these industrial sectors must meet strict standards of surface quality and dimensional accuracy.

Although most GFRP products are manufactured by special processes that provide the final shape [6,7], milling is necessary to achieve the quality requirements imposed for the parts. The milling process ensures both the imposed dimensional accuracy and the desired roughness of the machined surface [8]. Milling is particularly recommended for deburring hand lay-up parts to remove the excess material and imperfections that could affect the functionality of parts within an assembly. Numerous researchers have studied the phenomena that occur during the milling of GFRP parts, analyzing the factors that influence the geometric shape and dimensional tolerances of the final parts [7,9,10]. According to the study carried out by Hussein A. et al. [11], GFRP milling is essential in the automotive industry, especially for parts that will be integrated into complex assemblies, where the assembly precision requirements are extremely strict.

The characteristics of GFRP, such as the low thermal conductivity of the matrix, a low melting or burning point, and a high level of abrasion of the reinforcing material [12], lead to increased thermal phenomena during the milling process, which can adversely affect the quality of the machined surface and result in material accumulation on the tool. It is crucial to optimally select the cutting parameters to minimize the impact of these thermal phenomena on the surface quality. At the same time, the correct choice of tool geometry and cutting parameters contributes to maintaining low temperatures in the work area, an important aspect of milling polymer composites.

In recent years, there has been extensive research on thermal phenomena occurring in the cutting zone during the milling of metallic materials [13]. However, the effects of the thermal regime on GFRP milling have been much less addressed in recent studies. The complexity of this process continues to be a challenge for researchers and remains difficult to control in many industrial applications [2]. Milling GFRP involves major challenges such as fiber pullout, matrix burning [8], rapid tool wear, and damage to machine tools and equipment due to the abrasive nature of the chips. Also, the dust particles resulting from the chips are extremely dangerous for the health of people in the work area.

Over time, researchers have investigated the milling machinability of GFRP. Naresh et al. [14] analyzed the impact of various machining parameters, such as the fiber orientation angle, tool helix angle, cutting speed, and feed rate, on the machining forces and surface characteristics. Their study indicated that fiber orientation is the primary factor affecting surface roughness and cutting forces. In contrast, the feed rate has a smaller influence on these parameters in the GFRP milling process. The specialized literature indicates that elevated cutting forces result in a rise in temperature within the cutting zone. The authors assert that, during milling, both the cutting force and the temperature generated are critical factors affecting the final quality of the machined surfaces [7,15].

An experimental model for the optimization of milling parameters based on the Taguchi technique for GFRP was proposed by Jenarthanan et al. in [16]. Their study revealed that the fiber orientation angle (*p* = 66.75%) is the most statistically and physically significant parameter, followed by the feed rate (*p* = 15.05%), helix angle (*p* = 7.76%), and spindle speed (*p* = 0.30%). Khanna et al. [17] performed a comparative analysis of the influence of liquid carbon dioxide (LCO_2_) machining and dry machining on the cutting zone temperature in GFRP milling. They found that LCO_2_ reduced the temperature in the cutting zone by 88% compared to dry machining. The research by Shahabaz et al. [18] shows that the maximum cutting temperature is recorded at a fiber orientation of 90° in down milling.

Models for optimizing cutting parameters in GFRP machining, based on regression analysis, were proposed by some researchers in their works. The study conducted by Manoj N. et al. [19] shows that the cutting forces and temperature during GFRP milling are influenced by the depth of cut, feed rate, and cutting speed, each having a different impact on these parameters. The authors point out that a proper selection of parameters is essential to improve tool performance and increase the surface quality of machined parts.

According to Jasper et al. [20], the depth of cut and the material removal rate significantly influence the machinability of GFRP parts. The experimental results indicate that reducing the cutting speed and depth of cut can effectively improve the quality of the machined surface due to heat dissipation in the workpiece, tool, and chip, and it can also reduce the friction between the tool and the workpiece. Hanjie Hu et al. [21] studied the effect of milling cutting parameters on the quality of machined surfaces, noting that the use of milling cutters and twist drills at high speeds causes a rapid increase in temperature generated by friction, which aggravates the deterioration of machined surfaces. Hussein et al. investigated the impact of process parameters on the surface roughness of glass fiber composites and proposed an experimental model using ANOVA to optimize these parameters [11]. Junhao Ji et al. studied the impact of the cutting speed and feed rate on temperature, developing a mathematical model grounded in an empirical equation [15].

The milling of composite materials involves numerous challenges due to the abrasive nature of the reinforcing materials, which increases the temperature in the cutting zone, causes the rapid wear of the tools, and, consequently, compromises the quality of the machined surface [22,23,24]. The use of prediction models based on mathematical methods, such as neural networks, can contribute substantially to reducing the design time and to the optimal choice of process parameters for milling semi-finished products from polymer composite materials.

In this context, optimizing cutting parameters through a predictive neural network model is crucial for estimating the temperature in the cutting zone when milling GFRP parts.

In this paper, the authors aim to provide a model for predicting temperature in the cutting zone during GFRP milling using neural networks. The Artificial Neural Network model was developed with a virtual instrument made in LabVIEW 2024 software, a graphical programming language.

## 2. Materials and Methods

### 2.1. The Characteristics and Fabrication of Specimens

**Figure 1:**
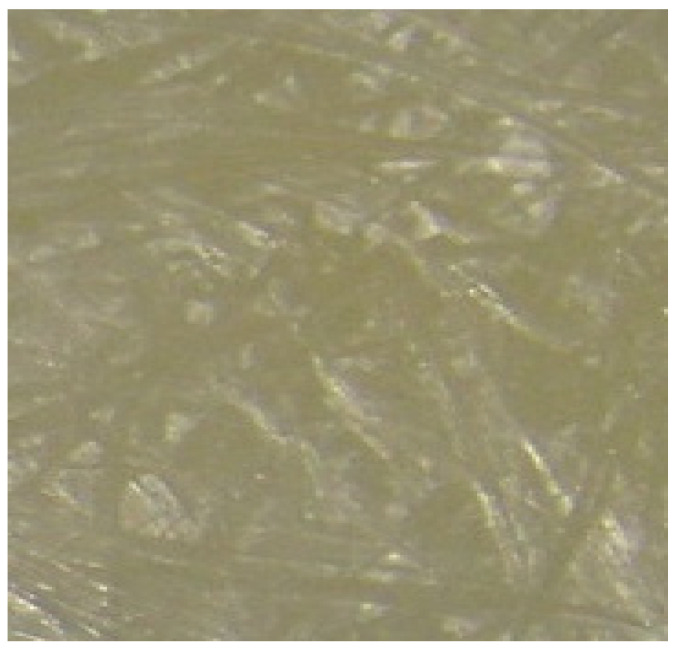
Laminated composite with polymer matrix randomly reinforced with glass fiber.

In this experimental study, we utilized specimens made from a polymer-based resin matrix reinforced with glass fibers. The composite materials studied consist of 60% AROPOL S 599 polyester resin randomly reinforced with 40% EC 12-2400 glass fiber (provided by Romturingia.SRL, Campulung, Romania). The workpieces (provided by Romturingia.SRL, Campulung, Romania) were fabricated using the spray lay-up technique in composite laminate form with dimensions of 200 mm × 200 mm × 3 mm, 200 mm × 200 mm × 6 mm, and 200 mm × 200 mm × 12 mm (Figure 1).

### 2.2. Raw Materials

AROPOL S 599 is an unsaturated polyester resin with a 34% styrene content frequently used in the composite materials industry. It has a suitable Brookfield viscosity (1100 mPas at 23 °C), which ensures the effective impregnation of glass fibers. After curing, AROPOL S 599 offers good mechanical strength, including a tensile strength of 55 MPa and a flexural strength of 100 MPa. In addition, the resin has good resistance to chemical agents, including moisture, with a water absorption of 28% in 24 h. AROPOL S 599 remains stable under conditions with temperatures of up to 125 °C. At temperatures higher than 125 °C, structural changes and thermal degradation occur in the material.

The glass fiber EC 12-2400 was used as a reinforcing material in the studied composite. The fiber has a filament diameter of 12 × 10⁻^6^ m and a density of 2.54 × 10^3^ kg/m^3^. This provides a tensile strength of 3450 MPa, a thermal conductivity of 1.3 W/(m·°C), and an elongation of 1.8–3.2%.

### 2.3. Equipment Description

The experimental tests were conducted in the laboratories of the National University of Science and Technology Politehnica Bucharest. The workpiece machining was carried out with a universal milling machine, FUS 22 (Romania, Oradea). The key technical specifications of the machine tool are as follows:

- A maximum longitudinal working stroke of 700 mm, a transverse measurement of 250 mm, and a vertical measurement of 370 mm;
- Vertical shaft speeds of 100, 160, 200, 250, 315, 400, 500, 630, 800, 1000, 1250, and 2000 rpm;
- Longitudinal advances of 12.5, 20, 31.5, 50, 80, 125, 25, 40, 63, 100, 160, and 250 mm/min.

**Figure 2:**
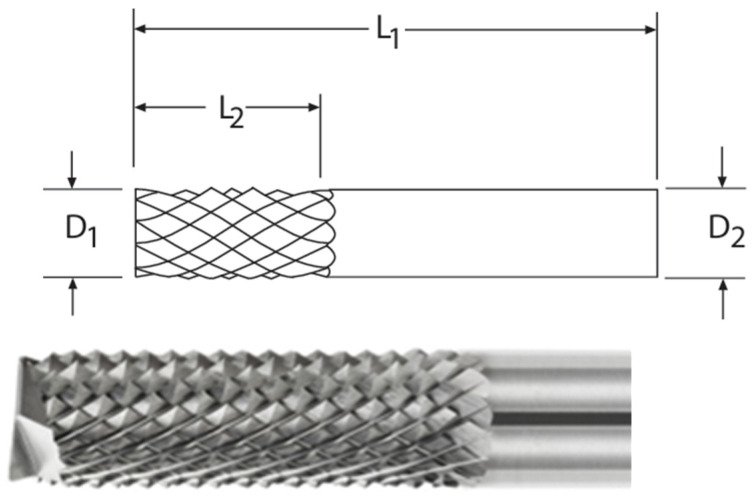
The cutting tool used for milling GFRP.

The cutting tools utilized in the experimental research were supplied by SGS Tools. The cutting tools have a special geometry and are recommended for GFRP machining. Ducobu, F. and collaborators performed a comparative analysis of various tool geometries used in milling GFRP composite material. The authors investigated the performance of different types of cutting tools and their effects on part material delamination and chipping forces. According to the study’s results, using these tools is an effective option for generating low forces and, implicitly, low temperatures in the cutting area [2]. Figure 2 shows the geometry of the tool used in the milling process of the GFRP parts.

Table 1 outlines the characteristics of the cutting tools employed in the milling process.

### 2.4. The Parameters of the Cutting Regime

The cutting parameters for the milling of GFRP were established based on the properties of the analyzed materials, the recommendations of the manufacturer of the cutting tools, and the technical specifications of the machine tool. The cutting parameters used as independent variables in the milling experiments included the cutting speed *vc* (m/min), feed rate *vf* (mm/min), and axial depth of cut *ap* (mm). The radial depth of cut was kept constant with the value of *ar* = 1 mm in each experimental run. Three levels were established for each parameter of the milling process (Table 2).

The factorial plan was developed using the central composite design method and comprises 15 experimental trials (Table 3).

In this experimental study, the infrared thermography method was used to determine the temperature of the cutting tool, the temperature of the workpiece, and the temperature of the chip. The temperature spectrum in the tool, workpiece, and chips was analyzed with a ThermaCAM SC 640 camera (provided by FLIR Systems AB, Danderyd, Sweden). The accuracy of temperature measurements using the infrared thermography method is influenced by several key factors, including material emissivity, ambient temperature, environmental humidity, and measurement distance. Additional factors include the angle between the surface and the thermographic camera, the camera’s spectral range, and its resolution. The emissivity of the composite materials analyzed typically ranges from 0.85 to 0.95. Using the settings menu of the ThermaCAM SC640, the emissivity was adjusted to match the material’s approximate theoretical value to ensure accurate measurements. Ambient temperature and humidity were recorded using a ThermoPro digital weather thermometer with a precision of ±1 °C and a humidity tolerance of ±2% *RH*. The room where the measurements were conducted had a temperature of 24 °C and a humidity level of 48% RH. The thermographic camera was placed 800 mm from the analyzed surface and positioned at a 60° angle relative to the surface. The ThermaCAM SC640 features a resolution of 640 × 480 pixels and an accuracy of ±2 °C. The analysis and interpretation of the acquired data were performed with ThermaCAM Researcher Professional 2.8 SR-2 software. The prediction model was developed using the ANN add-on toolkit NI Super Simple Neural Network in LabVIEW 2024 software, a graphical programming language.

## 3. Results

For this study, three tests were performed using identical cutting regime parameters for each experiment. The arithmetic mean of the three maximum measured temperature values was calculated, and the average maximum temperature values are presented in Table 4.

The measurements made during the milling of GFRP showed that the maximum temperature values were recorded in the contact zone between the tool and the machined surface, belonging to the range of [61.99 °C, 85.19 °C]. The lowest temperature was recorded for the first experiment, and the highest temperature for the eighth experiment. The minimum temperature value, *T* = 31.99 °C, was obtained for machining with a cutting speed of *vc* = 18.84 m/min, a feed rate of *vf* = 100 mm/min, and an axial depth of cut of *ap* = 3 mm. This low temperature was caused by the reduced contact area between the tool and the workpiece and the lower cutting speed, which generates less heat. The maximum value of the temperature (*T* = 85.19 °C) was recorded on the contact surface between the tool and the surface to be machined in the case of machining performed with a cutting speed of *vc* = 75.39 m/min, a feed speed of *vf* = 250 mm/min, and an axial depth of cut *ap* = 12 mm. This increase in temperature is due to the large contact surface between the tool and the workpiece where the abrasive character of the tool has intensified the frictional forces.

**Figure 3:**
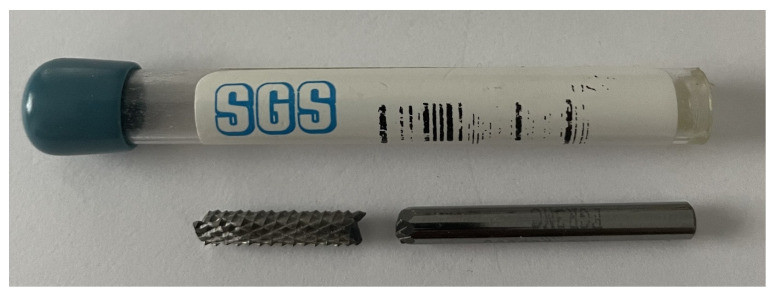
Broken cutting tool.

Before establishing the cutting parameters process for the factorial program of this study, several experimental trials were carried out with cutting speeds in the range of 150–250 m/min. During machining at high speeds, the temperature in the cutting area increased rapidly in a very short period, leading to structural transformations in the composite matrix, and it transitioned from a solid crystalline state to a liquid state. Additionally, at these elevated cutting speeds, the 3 mm diameter cutting tool broke due to the accelerated rise in cutting forces (Figure 3). This rapid increase in cutting forces was attributed to the melting of the matrix material, which caused molten material to deposit on the tool and adhere to the workpiece.

The adhesion of the matrix material to the cutting edge directly influenced the contact between the tool and the workpiece, causing changes in the generated and transferred heat in the three zones: the chips, workpiece, and cutting tool. In addition, due to the different thermal coefficients of the matrix and the reinforcing materials, internal stresses were formed between the fibers and the matrix, caused by the coefficient of expansions of the components, which led to the rupture of the interface between them.

The thermal phenomena generated during the milling process have a huge impact on the machined surface quality. Inadequately chosen parameters of the cutting parameters led to the melting of the material in the matrix, which, after solidification, generated a shape like the tool geometry on the surface. The high cutting speed had less impact on tool wear. Therefore, to reduce the negative effects in the milling process of the GFRP material, processing with cutting speeds lower than 100 m/min was chosen. During the processing of the studied materials, no liquid was used to reduce the temperature in the cutting zone. In the case of GFRP, the use of cooling liquids is not recommended due to the tendency of the polymer matrix material to absorb these liquids, which can affect the integrity and properties of the material [25].

In the milling process, the distribution of the heat generated by the mechanical work of cutting between the workpiece, tool, chip, and the surrounding environment depends on the cutting process, the thermal properties of the materials used for the part and the tool, as well as on the parameters of the cutting process. Experimental research for the up-milling of GFRP showed that a significant proportion of the heat generated was transferred to the tool, and only a small part was transferred to the piece and chips.

The temperature distribution in the milling tool was divided into three areas as follows:

- The area on the cutting surface of the tool. Here, the heat was generated by the deformation effort required to break the material.
- The area on the clearance surface. Here, the heat was generated by the friction between chips and the workpiece surface.
- The deformation zone. Here, the heat was generated by the friction between the tool and part.

In up-milling, the tool zone with the highest temperature was identified on the clearance face, near the cutting edge. This phenomenon is determined by the abrasive feature of the reinforcing material and the low thermal conductivity of the polymer matrix. The increase in the axial cutting depth led to the temperature increase because of the increase in the contact surface between the tool and the workpiece.

**Figure 4:**
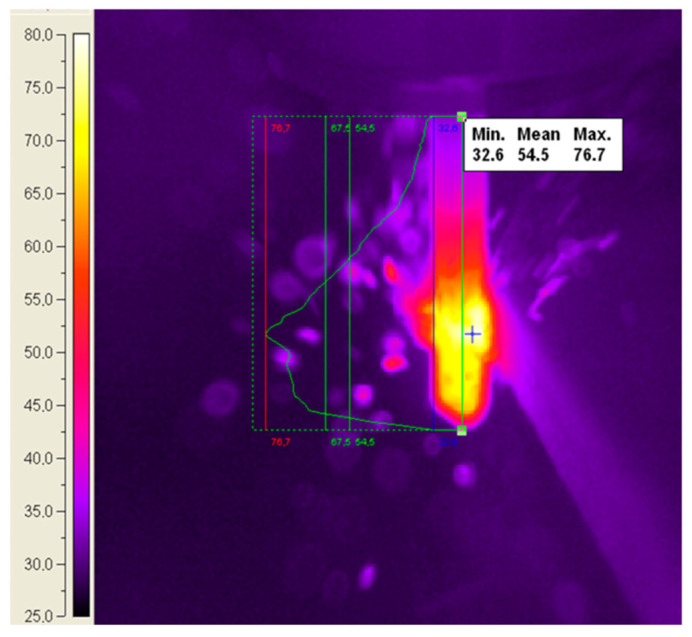
The temperature distribution along the axial direction of the cutting tool.

However, experiments have shown that the cutting speed influences the temperature in the cutting zone more significantly than the axial depth of the cut and the feed rate. The geometrical and constructive parameters of the cutter did not have a considerable impact on the temperature variation. Figure 4 illustrates the temperature variation curve along the axial direction of the tool.

Artificial Neural Networks (ANNs) have demonstrated superior performance in the prediction and optimization of machining processes when compared to traditional methods [26]. Their ability to model complex, nonlinear relationships between process parameters enables them to deliver more accurate and reliable predictions, making them highly effective in optimizing machining outcomes [27]. Previous studies have predominantly utilized ANN models in machining processes like turning processes (42.07%), followed by milling (34.48%) and drilling (23.45%) [28].

An Artificial Neural Network (ANN)’s structure and function are based on biological neural networks found in the human brain, and it represents a computational model in which there are layers with interconnected units called neurons or nodes, which process and transmit information [29,30]. The input layer receives the raw input data and passes them to the next layer. Hidden layers are intermediate layers that process the inputs received from the input layer by applying weights and activation functions to them. Multiple hidden layers can capture more complex patterns. The output layer produces the final result or prediction based on the processed information from the hidden layers.

**Figure 5:**
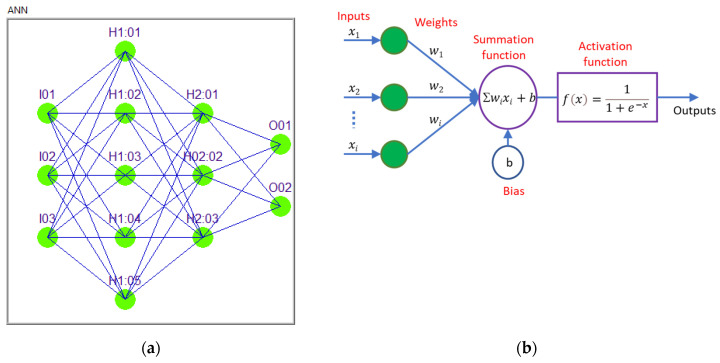
Artificial Neural Network (ANN) architecture. (**a**) ANN—general architecture; (**b**) neuron general mathematical representation model.

A general architecture representation is shown in Figure 5a. The input layer has three neurons, I01, I02, and I03; the first hidden layer contains five neurons, H1:01, H1:02, H1:03, H1:04, and H1:05; and the second hidden layer contains three neurons, H2:01, H2:02, and H:03. The output layer has two neurons: O01 and O02.

A general mathematical representation model of a single neuron from an ANN is shown in Figure 5b. The input vector (*x*_1_, *x*_2_, … *x_i_*) is passed through weighted connections, where each input *xi* is multiplied by its corresponding weight (*w_i_*). The resulting weighted sum is then combined with a bias term, b, and the final output is produced by applying an activation function to this sum.

In this case, we chose a feedforward neural network with a sigmoidal unipolar activation function which uses the sigmoid function to transform the input to the neurons at each layer, helping the network to capture nonlinear patterns and map inputs to outputs within a bounded range of 0 to 1 [31].

Resilient Propagation (RProp) was selected as the training algorithm for this ANN study due to its versatility and its ability to dynamically adjust the step size for each weight independently, making it well suited for a wide range of applications [32].

ANN Software for Temperature Prediction

The current ANN study tried to predict the maximum temperature *Tmax* (*C*) during the dry milling process for GFRP based on three key process parameters (independent variables): the cutting speed *vc* (m/min), feed rate *vf* (mm/min), and axial depth of cut *ap* (mm).

The software development and modeling of the ANN were carried out in LabVIEW, a system engineering software that enables the development of data analysis algorithms and custom engineering user interfaces using a graphical programming approach [28]. Leveraging Virtual Instruments (VIs) and employing data flow programming the ANN software involved the creation of modular subVIs and the integration of advanced programming techniques to efficiently handle complex data processing tasks. This approach enabled seamless functionality and scalability within the application, ensuring a robust and efficient system architecture.

The ANN add-on toolkit used was an NI Super Simple Neural Network based on a feedforward neural network with a unipolar sigmoidal activation function and the RProp training algorithm [33]. Sigmoid activation can capture nonlinear dependencies between inputs and outputs, and bounding the output in our case between 0.2 and 0.8 is essential for stable training in temperature prediction, where values are positive and limited within a certain range. RProp’s independence from the learning rate makes it advantageous in avoiding oscillations in training, which could be crucial given the variability in milling data.

Using the experimental datasets obtained from the actual analyzed milling GFRP process, an algorithm was designed using specific sub-VIs to preprocess the data for ANN training. This process generated tailored datasets for training and validation. To ensure optimal model performance, separate datasets were employed for the training, validation, and testing phases.

**Figure 6:**
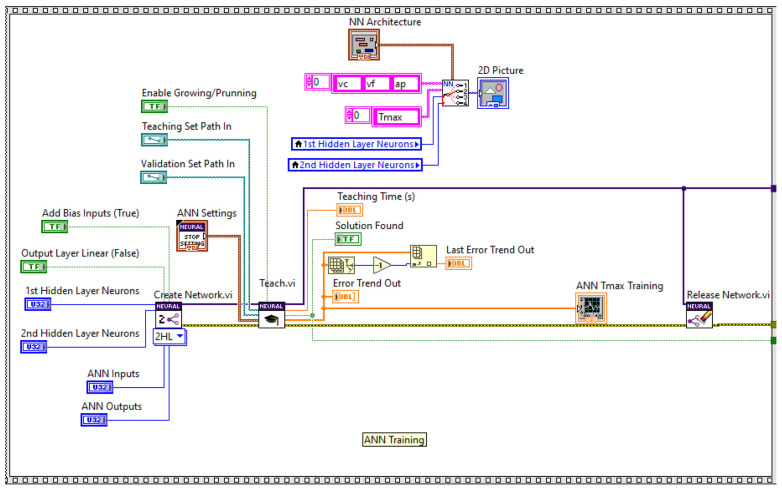
Partial diagram of ANN sub-VI used to create, teach, and test, and ANN.

The ANN training process starts by initiating a new ANN configuration by the sub-VI Create Network.VI with a specific set of parameters regarding the number of neurons for each layer: the input layer (ANN inputs), output layer (ANN outputs), the first hidden layer, and the second hidden layer. A bias of 1 is added to all neurons to improve the performance of the teaching algorithm. Then, the network starts the training with the sub-VI Teach.vi using distinct dataset files for teaching and validation based on a specific set of ANN training parameters. Figure 6 shows a partial diagram of the sub-VI code used to create, train, and test an ANN developed in LabVIEW.

The dataset was carefully prepared to prevent the need for retraining the ANN, starting from the acquired experimental data, which included maximum temperature measurements recorded for each trial.

**Figure 7:**
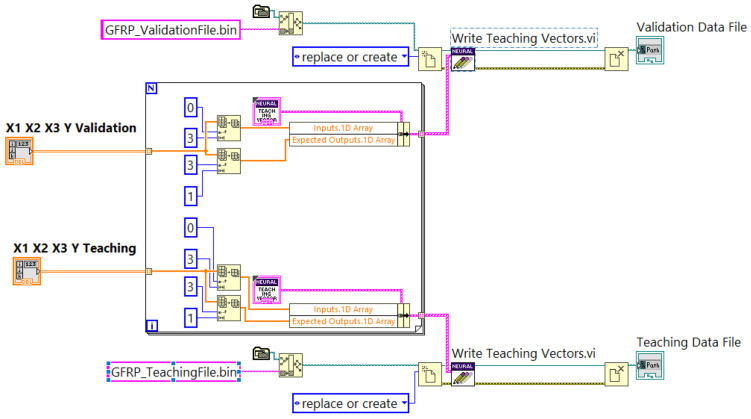
Partial diagram of sub-VI code for teaching and validating data files of ANN.

The ANN was trained using experimental datasets, with a separate dataset reserved for validation after the data had been normalized. Data preprocessing and normalization impact were required for generating the teaching and validation data files. Figure 7 illustrates how dedicated sub-VIs were used to generate these data files from the different datasets. *X*1, *X*2, and *X*3 represent arrays of values of the independent variables *vc* (m/min), *vf* (mm/min), and *ap* (mm), and they are provided for each teaching vector as Input 1D Array. Y is the array with values for dependent variable *Tmax*, and it is provided to the same teaching vectors as in Expected Outputs.1Array. The array of teaching vectors is then saved in specific data files for teaching and validating GFRP_TeachingFile.bin and GFRP_ValidationFile.bin.

Normalizing data improves model convergence, stabilizes learning, and impacts predictive performance, especially with heterogeneous data like the milling speed and feed rate, which vary significantly in scale.

Finally, the ANN dataset had to be organized in three different sets of files dedicated to teaching, validation, and testing.

A specialized search algorithm was applied to each dataset, with the ANN training parameters being adjusted within the following ranges:

- Max epocs: 10,000–20,000;
- Error goal: 2.396–2.4;
- Max time (s): 15–20 s;
- Error evaluation: over both sets;
- Vectors per iteration: 200–400.

Regarding configuration and hyperparameter tuning the reasoning behind iterative tuning, and the trial-and-error approach are essential due to the complexity of machining temperature prediction, which does not adhere to a one-size-fits-all configuration

**Figure 8:**
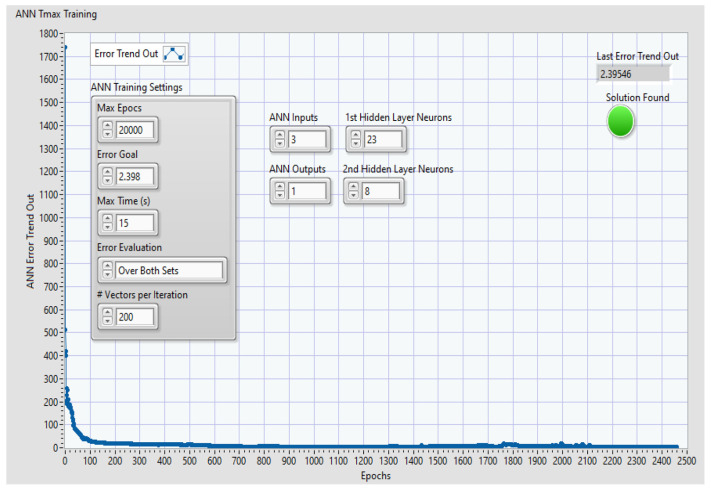
Example of plot of error trend out and key parameters for ANN 3:28:8:1 for GFRP.

According to Figure 8, the training process for each ANN architecture was based on specific key parameters like ANN training settings: the error goal, max time, training error evaluation, and vectors per iteration. The green LED displays “*Solution was found*” to indicate whether the algorithm validated or invalidated the training process and indicates the last value of “Error trend out” of the teaching process. For each time when the training process ended with “Solution found”, the corresponding ANN configuration data files were saved.

ANN Results: Analysis and Prediction

**Figure 9:**
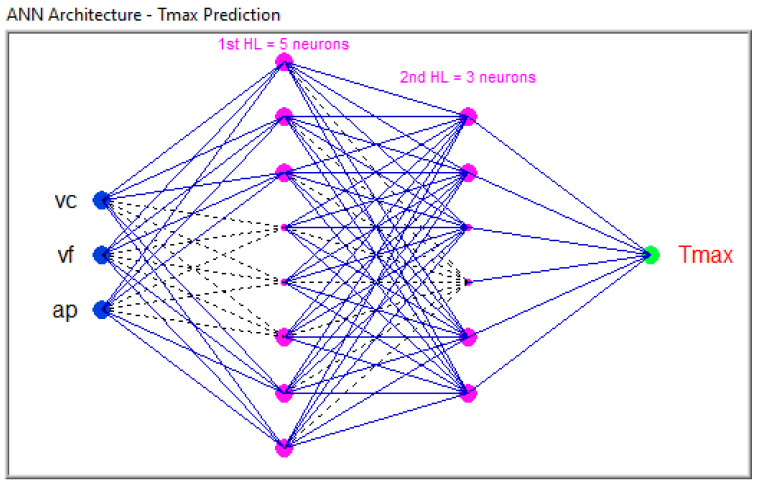
ANN 3:23:8:1 architecture for GFRP.

After the search algorithm identified several ANN architectures that met the error goal, their predictive performance was evaluated using a separate set of experimental data. Figure 9 shows an example of one of the ANN architectures deemed suitable.

**Figure 10:**
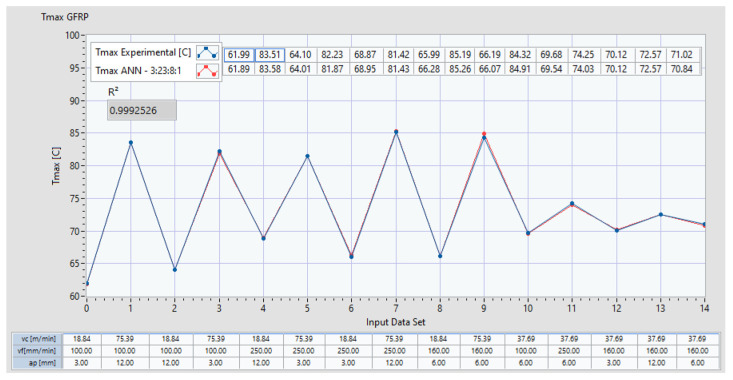
Comparison of *Tmax* between experimental data and ANN predictions with 3:23:8:1 architecture for GFRP.

Figure 10 represents the two plots of *Tmax*, one for the experimental dataset and the other for the predictions generated by the ANN (e.g., ANN 3:28:8:1, which corresponds to an ANN with three inputs, 28 neurons in the first hidden layer, and 8 neurons in the second hidden layer). The coefficient of determination *R*^2^ is shown, along with the maximum error (%) of the predicted data compared to the experimental results. Additionally, the input datasets are presented in the lower section of the plot.

**Figure 11:**
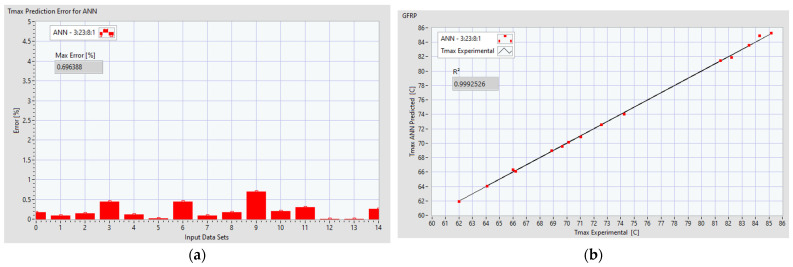
Prediction performance of *Tmax* for an ANN 3:28:8:1 for GFRP: (**a**) distribution of error per dataset; (**b**) coefficient of determination *R*^2^.

The performance of the ANN can be analyzed in Figure 11a, which presents the prediction error of *Tmax* for each dataset. Figure 11b shows the coefficient of determination *R*^2^ and the correlation between the predicted values and entire dataset.

**Figure 12:**
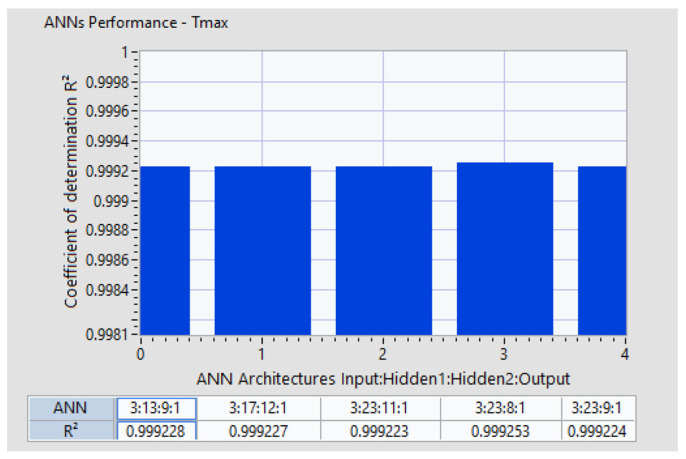
Coefficient of determination variation with ANN architectures.

We successfully identified and analyzed several ANN architectures that met the initial goal of achieving a prediction error of less than 2% and a coefficient of determination *R*^2^ greater than 0.9992. Figure 12 highlights 10 ANN architectures that satisfied these criteria for GFRP.

There are no universal guidelines for determining the optimal number of hidden layers in Artificial Neural Network architectures. This parameter can only be identified through a trial-and-error approach. [34]. The search algorithm succeeded in identifying different ANN architectures where neuron distributions are different for each hidden layer. For the case of GFRP, the architecture with ANN 3:23.8:1 has the best coefficient of determination, *R*^2^ = 0.999253, with three inputs (*vc*, *vf*, and *ap*) on the first layer, 23 neurons in the first hidden layer, 8 neurons in the second hidden layer, and one single output, *Tmax*. It was confirmed that multiple hidden layers enable the model to capture complex nonlinear relationships crucial for predicting outcomes in machining processes like milling.

**Figure 13:**
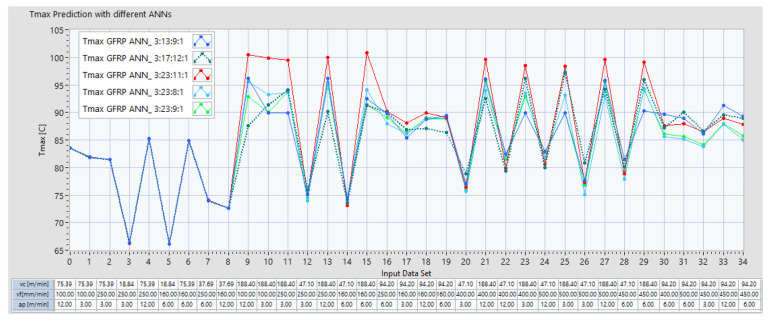
Top 5 ANNs for GFRP for the prediction of *Tmax* based on a dataset of 35 inputs.

With all five of the selected ANN architectures, another analysis was conducted by providing them with extended input datasets for analyzing temperature prediction behavior during the milling process. Figure 13 presents the prediction results for the five selected ANNs identified for the milling process of GFRP, all of which met the performance prediction criteria for the initial input data range. In this case, the input dataset was expanded with additional data corresponding to the following extended limits:

- *vc* (m/min): extending the maximum limit from 75.39 m/min to 188.4 m/min;
- *vf* (mm/min): extending the maximum limit from 250 mm/min to 500 mm/min;
- *ap* (mm): not extended.

Starting with dataset 9, the results indicate that for milling GFRP, the *Tmax* increases over 85 °C each time when the *vc* reaches 94.2 m/min, and it overpasses 95 °C when *vc* is 188.4 m/min. The variation in *vf* from 250 mm/min to 500 mm/min did not automatically increase the *Tmax.* For variations of *ap* = 3 mm, *ap* = 6 mm, or *ap* = 12 mm, the *Tmax* did not increase automatically. This was also confirmed by other similar studies, where it was shown that varying the feed per tooth (*fz*) and the depth of cut (*ap*) within the ranges analyzed did not produce a significant impact on the maximum temperature of the chips generated during milling [34].

The ANN prediction behavior of architecture ANN_3:23:11:1 indicates the highest values for *Tmax*, overpassing 100 °C for datasets 9, 10, and 15, but when starting with dataset 9, this architecture shows predictions quite different compared with the other architectures: ANN_3:23:8:1, ANN_3:23:9:1, ANN_3:13:9:1, and ANN_3:17:12:1. Consequently, this opened up a new research direction, prompting additional experimental trials where the milling process parameters would be varied within the following extended ranges: a cutting speed *vc* of 200–250 m/min, *ap* of 0.75 to 1 mm, *vf* of 500–600 mm/min, and *ap* of 12–14 mm. Higher values for specific parameters (e.g., the cutting speed) led to more complex patterns that the ANN had to learn, impacting architecture choices to manage the increased complexity. These extended ranges aim to explore the effects of broader parameter settings on the milling process outcomes. Also, we may consider potential ANN architectural adjustments (e.g., adding more neurons in specific layers) or alternative architectures (e.g., convolutional layers for spatial data) to improve prediction under new conditions.

## 4. Conclusions

Milling glass fiber-reinforced composite materials are necessary to remove excess material and other imperfections that can affect the functionality of the parts in an assembly. Dimensional tolerances imposed on the parts can be obtained if the temperature in the cutting zone does not exceed the temperature at which the polymer matrix remains stable. In this study, through the appropriate choice of parameters of the cutting regime, the temperature of 125 °C was not exceeded, which is the temperature at which structural changes and thermal degradation would have occurred in the material.

Milling polymer composites presents several problems and challenges due to the properties of the constituent materials.

As manufacturers begin to work with more advanced composite materials, the adaptability of ANN models becomes a competitive advantage. This study’s approach to optimizing GFRP milling can be extended to other fiber-reinforced composites by adjusting the ANN architecture to account for different mechanical properties, allowing manufacturers to expand their capabilities in machining newer composites.

The use of optimized cutting parameters as well as the choice of tools with specific GFRP geometry contribute to minimizing the phenomenon of excessive heat generation in the cutting area. For this study, low cutting speeds and appropriate feeds were adopted to avoid melting or damaging the polymer matrix and to avoid material deposits on the tool. The ANN model provides manufacturers with flexibility in customizing milling parameters to meet specific requirements for high-performance applications, such as aerospace and defense, where GFRP components must meet strict tolerance and durability standards. By tailoring parameters to optimize temperature and reduce microstructural damage, manufacturers can meet these stringent requirements with confidence.

In conclusion, the optimization of the milling parameters and the use of appropriate tools are essential for obtaining optimal results.

The ANN application software developed in this study demonstrated to be fully prepared for expansion and integration into larger subsystems, including modern cyber-physical systems. It is designed to assume predictive and adaptive functions, particularly in designing future products using materials such as GFRP composites, enhancing its role in advanced manufacturing environments.

In line with advancements in more powerful and efficient AI hardware, another key direction for software development is to expand ANN models, architectures, and AI programming techniques to accommodate additional relevant process parameters, such as tool displacement, vibration, and wear. By incorporating a greater number of independent variables as inputs to the ANN, the model’s predictive accuracy can be significantly enhanced, allowing for more precise and reliable predictions in complex processes.

The research results regarding the optimized milling process for GFRP are valuable for various applications in the automotive, aerospace, and consumer goods industries. Companies that process GFRP by milling can apply these findings to increase production efficiency by reducing the time needed to choose the optimal cutting parameters, minimizing the costs generated by defective parts and avoiding esthetic problems caused by melting and the solidification matrix generated by the high temperature. Temperature control in the milling zone also brings significant benefits, such as reduced cutting tool wear, increased tool life, and decreased tool change frequency, leading to lower operating costs. By optimizing the GFRP milling process, manufacturers can reduce material waste due to more precise process control, thereby helping to reduce the environmental impact and reduce the carbon footprint of the process.

## Figures and Tables

**Table 1:** Characteristics of cutting tools.

Tool	Diameter *D*1 mm	Length *L*2, mm	Diameter *D*2, mm	Length *L*1, mm
FGR-2M	3	12	3	38
FGR-5M	6	19	6	50
FGR-9M	12	25	12	75

**Table 2:** The independent variables of the process parameters.

Cutting Parameter	Symbols	Level		
		−1	0	1
cutting speed	*vc * m/min	18.84	37.69	75.39
feed rate	*vf * mm/min	100	160	250
axial depth of cut	*ap * mm	3	6	12

**Table 3:** The factorial plan.

Experiment No.	Process Input Variables		
	*vc * m/min	*vf * mm/min	*ap * mm
1.	−1	−1	−1
2.	+1	−1	+1
3.	−1	−1	+1
4.	+1	−1	−1
5.	−1	+1	+1
6.	+1	+1	−1
7.	−1	+1	−1
8.	+1	+1	+1
9.	−1	0	0
10.	+1	0	0
11.	0	−1	0
12.	0	+1	0
13.	0	0	−1
14.	0	0	+1
15.	0	0	0

**Table 4:** The maximum values of the measured temperatures.

Experiment No.	Process Input Variables			Temperature
	*vc* (m/min)	*vf* (mm/min)	*ap* (mm)	*T* (°C)
1.	18.84	100	3	61.99
2.	75.39	100	12	83.51
3.	18.84	100	12	64.1
4.	75.39	100	3	82.23
5.	18.84	250	12	68.87
6.	75.39	250	3	81.42
7.	18.84	250	3	65.99
8.	75.39	250	12	85.19
9.	18.84	160	6	66.19
10.	75.39	160	6	84.32
11.	37.69	100	6	69.68
12.	37.69	250	6	74.25
13.	37.69	160	3	70.12
14.	37.69	160	12	72.57
15.	37.69	160	6	71.02

## Data Availability

Data are contained within the article.
